# Association of Genetic Variants, Such as the μ-Opioid Receptor 1 (*OPRM1*) *rs1799971* and Catechol-O-Methyltransferase (*COMT*) *rs4680,* with Phenotypic Expression of Fibromyalgia

**DOI:** 10.3390/biomedicines13051183

**Published:** 2025-05-13

**Authors:** Isabel Erenas Ondategui, Julia Gómez Castro, Sandra Estepa Hernández, Celia Chicharro Miguel, Regina Peiró Cárdenas, Ana Fernández-Araque, Zoraida Verde

**Affiliations:** 1 Faculty of Health Sciences, University of Valladolid, 42004 Soria, Spain; isabel.erenas@uva.es; 2 Department of Nursing, Faculty of Health Sciences, University of Valladolid, 42004 Soria, Spain; juliamaria.gomez@uva.es (J.G.C.); anamaria.fernandez@uva.es (A.F.-A.); 3 Department of Biochemistry, Molecular Biology and Physiology, Faculty of Health Sciences, University of Valladolid, 42004 Soria, Spain; sandra.estepa@uva.es (S.E.H.); celia.chicharro@uva.es (C.C.M.); 4 Centro de Estudios Gregorio Marañón, Fundación Ortega-Marañón, 28010 Madrid, Spain; 5 School of Medicine and Health Sciences, Tecnológico de Monterrey, Guadalajara 45201, Mexico; a01635566@tec.mx; 6 Grupo Investigación Reconocida (GIR) Farmacogenética, Genética del Cáncer, Polimorfismos Genéticos y Farmacoepidemiología, University of Valladolid, 47005 Valladolid, Spain; 7 Unidad de Investigación Consolidada (UIC) de Castilla y León 387, University of Valladolid, 42004 Soria, Spain

**Keywords:** genetic variants, fibromyalgia, COMT, OPRM1, fatigue, severity symptoms, borg scale, chronic pain, gender

## Abstract

**Background/Objectives**: Genetic variants, such as the µ-opioid receptor 1 (*OPRM1*) *rs1799971* and the catechol-O-methyltransferase (*COMT*) *rs4680*, have been considered among the potential causes in the development of some chronic pain conditions. In this regard, there are controversial results regarding their roles in fibromyalgia (FM). We aimed to investigate whether the OPRM1 rs1799971 and COMT rs4680 polymorphisms are associated with the development of or susceptibility to FM, as well as their potential association with syndrome characteristic variables, in a sample of the Spanish population with and without FM. **Methods**: The present study analysed *COMT* Val158Met and *OPRM1* Asn40Asp genetic variants in 311 FM patients (301 women and 10 men) and 135 non-FM participants (120 women and 15 men). In addition to clinical variables, widespread pain index (WPI), symptom severity scale (SSS) (fatigue, rest quality, and cognitive symptoms), pain, stress episodes, and Borg scale were collected. **Results**: The main results indicate that women carrying the Val/Val genotype (i.e., high COMT activity) exhibited significantly lower levels of fatigue, cognitive impairment, and total SSS than heterozygote carriers. In addition, Met allele carriers (i.e., lower COMT activity) showed higher probabilities of suffering a stress episode and higher levels of exertion during daily activities. **Conclusions**: The present research suggests a link between dopaminergic dysfunction and exacerbated, frequently described symptoms in female FM patients. Although further research with wider genetic variants and recruited patients is needed, these results point out the necessity of considering gender as a separate category in chronic pain studies.

## 1. Introduction

Fibromyalgia (FM) is an increasingly common syndrome (prevalence of 2–4% worldwide) that predominates among women and typically manifests between the ages of 30 and 35 years [1]. In general terms, this syndrome is characterized by widespread chronic musculoskeletal pain, frequently accompanied by fatigue, cognitive dysfunction, a variety of somatic complaints, and psychiatric disturbances, such as depression and anxiety [2,3]. The disorder involves pain dysregulation and is influenced by genetic, environmental, and neurobiological factors [1,4], specifically diffuse hyperalgesia (increased pain in response to typically painful stimuli) and/or allodynia (pain in response to typically non-painful stimuli) [5,6,7,8].

Despite advances in understanding the underlying pathological processes, the use of quantitative biomarkers for the assessment and diagnosis of FM remains a gap in clinical practice, with up to 75% of people with this syndrome remaining undiagnosed [9]. Etiopathogenesis is still unclear, as various variables seem to play a significant role in the development of this syndrome, including psychosocial factors, environmental factors, and genotype [10,11].

Genetic factors have been considered among the potential causes in the development of FM. In this regard, µ-opioid receptor 1 (*OPRM1*) *rs1799971* and catechol-O-methyltransferase (*COMT*) *rs4680* polymorphisms have been proposed to be associated with FM [12]. The *OPRM1 rs1799971* polymorphism causes an amino acid substitution at position 40 in the first exon of the OPRM1 protein, changing asparagine (Asn) to aspartate (Asp). This variation in the allele, whether Asn or Asp, affects the protein’s affinity for β-endorphin, suggesting a possible mechanism by which this polymorphism could influence pain sensitivity and affect frontoparietal network activity during pressure stimulation in individuals with FM [13,14,15]. *COMT* is the most extensively researched gene in relation to pain [14]. As one of the main enzymes responsible for deactivating catecholamines, such as dopamine, the Val158Met *rs4680* polymorphism causes the substitution of valine (Val) with methionine (Met) at codon 158 in the *COMT* gene [16,17]. This substitution leads to reduced activity of the encoded enzyme, affecting dopamine availability in the brain [16,18]. Homozygous carriers of the Met allele have demonstrated increased pain sensitivity in FM patients compared to those carrying the Val allele [18,19].

However, although some studies have demonstrated that the *COMT rs4680* variant increases the risk of developing FM syndrome [19], other investigations have shown no association [20,21]. Therefore, further research is needed to resolve these discrepancies and to better understand the role of *rs4680* and *rs1799971* polymorphisms in FM development. In addition, the studies developed have conflicting results on the prevalence of genetic variants in FM patients.

The aim of this work was to investigate whether the *OPRM1 rs1799971* and *COMT rs4680* polymorphisms are associated with the development or susceptibility to FM, as well as their potential association with syndrome characteristic variables, in a fully phenotyped sample of the Spanish population with and without FM.

## 2. Materials and Methods

### 2.1. Participants

A prospective observational cross-sectional study was conducted between 2022 and 2024 in a sample of unrelated patients with diagnoses of FM. In addition, we included a representative cohort of non-FM healthy participants matched by age and gender to the FM cohort. All FM participants were recruited from different associations of patients with FM (AFINSYFACRO, FIBROPARLA, AFICRO-Vall, AFACYL, and FIBROAS FM Associations), and the non-FM cohort were recruited consecutively in primary care units of the Soria Health Area.

Intentional and voluntary sample selection was carried out, and 322 FM patients older than 18 years old were initially recruited, although 11 patients were excluded because of nonalignment with the inclusion/exclusion criteria or withdrawal of participation in the study. FM patients should have been diagnosed according to the American College of Rheumatology (ACR) 2016 criteria. Exclusion criteria included cognitive impairment due to any type of neurological disease or disorder, psychosis, any type of oncological condition, pregnancy, and being institutionalized. In addition, individuals with infusion pumps for chronic pain management were excluded. Finally, 135 healthy controls were included in the study (of the 136 recruited controls, one was excluded due to a family history of FM).

The study was approved by the Valladolid Este Ethics Committee (PI-GR-21-2418) and followed the Helsinki Declaration and data protection law. All participants signed a written informed consent form with an explanation of the study and the option to withdraw their participation at any time.

### 2.2. Patient-Reported Outcome Measures (PROMs)

All participants were phenotyped, and they completed standardized and validated health and pain status questionnaires through a clinical interview in which a research nurse recovered several variables in addition to demographic data. All the patients also completed data about their family history of FM, medication, stress episodes, and rest quality.

#### 2.2.1. Pain Outcomes

For the analysis of pain status, the widespread pain index (WPI) and the visual analogue scale (VAS) were used. The WPI measures the presence of bodily pain in 19 regions of the body during the last 7 days. One point is added for each area of the body where the patient reports pain. Higher scores indicate greater pain spread [22].

The VAS is a simple and frequently used method for the assessment of variations in the intensity of pain [23,24].

#### 2.2.2. Symptom Severity

All participants completed the Symptom Severity Scale (SSS). This scale measures three dimensions, focusing on fatigue levels (FSS), waking unrefreshed (WunSS), and cognitive symptoms (CoSS), with scores ranging from 0 (asymptomatic) to 3 (severe symptoms) [25,26].

In addition, we analysed the FM Severity Scale, adding the scores of SSS (score 0–3) and WPI (score 0–19), resulting in a new score between 0 and 32 [27,28].

#### 2.2.3. Borg Scale

The Borg scale rates perceived exertion (RPE) and is a valid tool for monitoring exercise intensity in FM [29]. The 10-point Borg CR-10 scale ranges from 0 (“nothing at all”) to 10 (“very, very strong”) [30]. During the interview, the research staff presented the CR-10 scale on a white sheet of paper, and the participants pointed at the number representing the overall exertion perceived during their activities of daily living (ADLs).

### 2.3. DNA Collection and Genetic Analysis

DNA was extracted from white blood cells from plasma in EDTA, using the E.Z.N.A.^®^ Tissue DNA Kit (V-Spin, Omega Bio-Tek, Norcross, GA, USA; REF. D3396-02). These DNA samples were frozen at −20 °C until their use for genotyping. An RT-PCR was performed with an Applied Biosystems StepOne™ Real-Time PCR System Thermal Cycling Block using TaqMan™ SNP Genotyping Assay (Applied Biosystems, Foster City, CA, USA) to genotype *COMT* allele *2 (*rs4680*; ID: C__25746809_50) and *OPRM1 rs1799971* (ID: C___8950074_1_) polymorphisms. After this assay, *COMT* genotypes were designated as G/G for Val/Val homozygote, as A/A for Met/Met homozygote, and as G/A for Val/Met heterozygote. On the other hand, *OPRM1* genotypes were named as A/A for Asn/Asn homozygote, G/G for Asp/Asp homozygote, and as A/G for Asn/Asp heterozygote. Alleles’ dichotomization was carried out by defining a dominant model.

### 2.4. Statistical Analysis

The statistical analysis was conducted using SPSS Statistics 29.0 (SPSS Inc., Chicago, IL, USA). Descriptive statistics were presented as mean ± standard deviation for continuous variables and as frequencies and percentages for categorical variables. Assumptions of normality were verified for continuous variables, and appropriate transformations were applied where necessary to meet the test assumptions. For the analysis of demographic variables and variables related to pain and symptoms, quantitative variables were compared between groups using the Pearson correlation coefficient, while categorical variables were analysed with the Chi-square test. Moreover, quantitative and categorical variables were contrasted with Student’s *t*-test. In addition, logistic regression (LR) analysis was performed to examine potential interactions between *COMT* and *OPRM1* genotypes and phenotypes in crude and adjusted models. Results were considered statistically significant at a *p*-value < 0.05. Deviation from the Hardy–Weinberg equilibrium for the *OPRM1* and *COMT* polymorphisms was tested by the Chi-squared test.

## 3. Results

### 3.1. Demographic, Clinical, and Phenotype Analyses

The demographic and PROM profiles showed significant differences between the two groups (Table 1). Notably, FM patients were slightly older on average (56.77 ± 8.24 years, range 36–85 years) than non-FM participants (51.38 ± 14.18 years, *p*< 0.001, range 19–84 years), and, as expected, female participants were predominant in both groups (96.80% vs. 88.90%, *p*< 0.001). Differences were also observed in medication: polypharmacy was significantly higher among FM patients (58.90% and 70.00%, women and men, respectively) compared with non-FM peers (0.00% in both groups), being the most prevalent analgesics. As expected, in both male and female groups, a comparison of FM with non-FM participants showed statistically significant differences in FSS, WunSS, CoSS, total SSS, rest quality, stress episode, and Borg scale (see Table 1). In the case of VAS, we only observed statistically significant differences between FM and non-FM peers in the female category (*p* = 0.006) (Table 1).

In addition to the diagnosis of FM following the American College of Rheumatology (ACR) 2016 criteria, we also evaluated the WPI. In accordance, FM patients had a mean score indicating elevated extensive pain (11.12 ± 3.94) and (11.20 ± 3.93) in women and men, respectively (Table 1).

Pearson correlation analysis was performed among symptom scales and demographic variables. In addition to expected correlations between scales, we also observed, in the group of women, a positive correlation between age and total SSS, FSS, WunSS, CoSS, and sleep quality (coefficient = 0.195, *p* < 0.001; coefficient = 0.192, *p* < 0.001; coefficient = 0.154, *p* = 0.002; coefficient = 0.191, *p* < 0.001 and coefficient = 0.182, *p* < 0.001, respectively) and a negative correlation between age and stress episode (coefficient = −0.197, *p* < 0.001). In the case of the men’s group, we only observed a positive correlation between age and WunSS (coefficient = 0.396, *p* = 0.050).

### 3.2. Genetic Polymorphisms: Distribution and Frequencies

The frequencies of the analysed genetic polymorphisms are shown in Table 2. The genotype distribution for *COMT rs4680* and *OPRM1 rs1799971* polymorphisms did not deviate from that expected based on the Hardy–Weinberg equilibrium (*p* = 0.502 and *p* = 0.288) and (*p* = 0.580 and *p* = 0.212) for the non-FM and FM groups, respectively. We found only a marginal difference in the genotype distribution of *COMT rs4680* and *OPRM1 rs1799971* between the groups (FM men and non-FM men), with the alleles A472 and A118 more prevalent in non-FM males (Table 2).

### 3.3. COMT rs4680 and OPRM1 rs1799971 Polymorphisms: Impact in Relation to Phenotype

Next, LR analyses were performed to study the association between genetic polymorphisms and the phenotypes of the participants, stratified by gender (Table 3 and Table 4).

In women, a significant relationship between FSS, WunSS, total SSS, and *COMT rs4680* polymorphism was found, as 158GG (Val/Val) genotype carriers presented a better status than 158A (Met) carriers (Table 3). In contrast, the *COMT* 158A (Met) allele was found to increase the probability of suffering a stress episode and of increased effort on the Borg scale (*p* = 0.005 and *p* = 0.001, respectively). In contrast, LR did not reveal any significant difference in phenotype depending on *OPRM1 rs1799971* in the women’s group.

On the other hand, in the male population, we observed several significant associations between *OPRM1* polymorphism and FSS, WunSS, total SSS, and rest quality. *OPRM1 rs1799971* A/A (Asn/Asn) genotype carriers presented a better status in PROMs than G (Asp) carriers (Table 4).

## 4. Discussion

FM is defined as a widespread chronic pain syndrome associated with fatigue, sleep disturbance, cognitive impairment, affective disorder, and somatic symptoms. Although conceptually clear, the wide variety of procedures used in phenotyping FM patients makes it difficult to compare results across genetic association studies and to obtain solid conclusions. Moreover, most genetic association studies do not describe these parameters as being clearly linked to the representativeness of the sample.

In this study, in accordance with the definition, FM patients (male and female) presented significantly higher levels of fatigue, cognitive impairment, and less restorative sleep than their non-FM peers. These findings are consistent with previous research that emphasizes the pervasive role of non-restorative sleep and the prevalence of moderate cognitive impairment in FM pathophysiology [31,32]. Self-reported cognitive decline has also been correlated with reduced functional ability in daily life, although its association with objective cognitive tests remains controversial. These cognitive impairments may reflect broader distress rather than discrete cognitive dysfunction, as previously suggested by analyses of subjective and objective measures of FM cognitive performance [28,31].

The elevated WPI score (11.12 ± 3.94) and the elevated SSS score (5.73 ± 1.14) in total FM patients (male and female) reinforce the central role of chronic widespread pain in the diagnosis and impact of FM. The score is likely to summarize both pain severity and multidimensional symptom burden, as evidenced by its strong association with fatigue, sleep disturbance, and cognitive dysfunction [31]. In addition, the FM diagnostic criteria introduced in 2010, which include the combined scores of the SSS and WPI, have been developed and evaluated as a more effective diagnostic framework compared with the earlier tender-point-based classification system originally established by the ACR, finding that a WPI ≥7, combined with high SSS, identified FM patients according to their modified ACR 2010 criteria [25,26]. With regard to analysing effort during daily activities, the Borg scale was measured. The FM participants presented higher levels of exertion than the non-FM participants. These data were correlated with VAS and rest quality, showing higher pain sensitivity and sleepiness in women with FM. Morishita et al. also studied the relation between the Borg scale and pain, finding significant relationships between these two factors [33].

This study examined the role of two genetic polymorphisms in *OPRM1 rs1799971* and *COMT rs4680*, commonly related to chronic pain, within the clinical phenotype in women and men with and without FM.

The allele frequencies observed for both genetic variants in women were similar to frequencies previously reported in Caucasians and the Spanish population [14,17,18,34]. In the case of the male population, the distribution was different, probably due to the low number of participants studied.

The current results reveal that, in the main, different genotypes of the Val158Met *COMT* genetic variant might contribute to individual differences in phenotyping features related to chronic pain in females. The *rs4680* genetic variant in the *COMT* gene has been previously studied in relation to multiple diseases and pain. The majority of studies on FM have reported heterogeneity in results [19,34,35,36,37]. The present results show that women carrying the Val/Val genotype (i.e., high COMT activity) exhibited significantly lower levels of FSS, WunSS, and total SSS than heterozygote carriers. In addition, Met allele carriers (i.e., lower COMT activity) showed higher probabilities of suffering a stress episode and higher levels of exertion during daily activities.

Our findings are in alignment with several previous studies supporting the idea that the *COMT* gene may influence fatigue perception in women with FM or in healthy participants [36,38,39]. Some authors have suggested that a reduction in COMT enzyme activity (i.e., the Met/Met genotype) is associated with a reduction in the density of endogenous opioid receptors, thereby increasing fatigue perception enhanced by pain sensitivity [39,40]. Moreover, lower COMT activity has also been related to an increase in cytokine response in animal models [41]. Cytokines cause hyperexcitability in pain transmission and the release of excitatory neurotransmitters, resulting in increased fatigue perception [42,43]. Both factors support the role played by the *COMT rs4680* genetic variant in the variability in fatigue symptoms.

Since COMT has a crucial role in metabolising dopamine, it was suggested that the common functional polymorphism in the *COMT* gene impacts sleep–wake regulation, and potentially sleep pathologies, with contradictory results [34,44,45,46]. As we mentioned, we observed that in the women that were analysed, the homozygous G/G (Val/Val) carriers presented better quality of sleep than the A (Met) carriers. To check the second item of total SSS (WunSS), we also measured the quality of rest in an independent way, and we obtained the same results. They seem consistent with previous evidence because individuals homozygous for the Val allele show higher COMT activity and lower dopaminergic signalling in the prefrontal cortex (PFC) than subjects homozygous for the Met allele [44]. Examining total SSS, we also observed a relationship between lower values and the Val158Val *COMT* genotype. Our results appear to indicate that the Val/Val genotype is associated with a lower severity of symptoms, similar to previous studies [20].

In addition, a significant relationship between the Val158Met *COMT* genetic variant and stress episode and level of exertion was found. Met158 *COMT* allele carriers, particularly women, presented higher probabilities of suffering a stress episode and higher perceived exertion during activities of daily living. Similarly, previous studies have reported that Met158 carriers are more sensitive to stress and exhibit higher anxiety and reactivity to lower levels of stress [44,47,48]. The Met allele probably leads to enhanced catecholinergic activity and might therefore impact cortical function, resulting in a stronger response [49]. Related to the Borg scale, we have found no published research in relation to COMT in FM. The Borg scale is a scale for RPE. It is a tool for estimating effort and exertion, breathlessness, and fatigue during physical work and is related to pain and fatigue. Accordingly, we have also observed a statistically significant positive correlation between Borg scale and VAS or FSS (*p* < 0.001, R = 0.175 and *p* < 0.001, R = 0.320). There is a possible overlap between peripheral pain perception and perceived exertion during exercise, resulting in an increase in sensory stimuli from the musculoskeletal system and, consequently, in RPE [50]. Borg scale scores could be used as an additional parameter for prescribing exercise intensity in aerobic exercise protocols, and the scale is a valid and moderately reliable tool for monitoring exertion intensity in women with FM [29].

Interestingly, we have found no relationship in the men’s group. COMT enzyme activity is reduced epigenetically by estrogens, with a 30% decrease in activity in females compared to males [51]. It is possible that this is related to the elevated effect in women who have both the Met158 allele and lower levels of COMT activity. Another possible explanation is the small number of male participants recruited in this group—not enough to achieve any kind of association.

On the other hand, we observed no relationship between the COMT rs4680 genetic variant and pain in either FM or non-FM pairs. As mentioned in the Results section, our data showed that patients with FM had worse VAS scores than non-FM patients, in accordance with previous studies, although the levels of self-reported pain in our cohorts are lower than in the aforementioned works. In our FM cohort, 72.8% of the participants presented mild pain levels. We think the fact that the patients recruited were not primary FM diagnosed, and had their pain well under control with the use of guided medication, may have influenced our results. As mentioned in the description of the study, we registered the medications they took, and the prevalence of analgesic use was about 74%.

In the case of the *OPRM1* gene, we observed no relationship between the A118G *OPRM1* genetic variant and any PROMs studied in the female group (see Table 3). Previous studies found similar results regardless of A118G variant presence [15,52]. However, other authors have reported a relationship between the G/G genotype of the *rs1799971* and pain ratings or pain catastrophizing [14,53,54]. In men, we observed a relationship between the G/G genotype and worse results in terms of SSS items, WunSS, or the suffering of stress episodes (see Table 4). However, due to the low number of male patients, we could not interpret the aforementioned data reliably. Because FM is more common in women, most studies include only female participants. Therefore, our results cannot be compared.

To the best of our knowledge, this study is the largest cohort study of well-characterized FM patients, including total SSS and level of exertion (Borg scale), where the *COMT* Val158Met and *OPRM1* Asn40Asp genetic variants are analysed and gender considered as a separate category despite the small number of men recruited. Compared with previous studies, our larger sample size is a strength of the present study [34,36,54,55]. In the main group, we used a balanced sample composed of women to better control gender differences. Previous studies have reported that approximately 25% of diagnosed FM patients recruited did not satisfy the ACR classification criteria, although they were considered to have FM by their physicians [25]. In our study, in addition to the ACR classification, we have included WPI and SSS in order to verify the diagnosis and to provide a classification based on severity assessments. We suggest using both methods to quantify FM symptom severity as a workable solution to this problem.

On the other hand, some limitations of our study may have affected our results. First, previous studies have suggested that hyperadiposity states may negatively modulate the clinical severity in FM. Thus, body composition must be part of the global assessment of FM patients [56]. Well-phenotyped patients, larger samples, and subgroup analyses are desirable, given the complexity of FM syndrome. Additionally, we have analysed only two genetic variants, so the inclusion of more genetic variants could be genetically more informative. The relationship between genetic variants and the symptoms of chronic pain diseases is complex. Genetic factors do not act in isolation; they interact with many other genetic and environmental factors that can increase the risk of more severe symptoms [37].

## 5. Conclusions

To conclude, our results suggest links between dopaminergic dysfunction and FM symptoms. Women carrying the *COMT* 158Met allele reported higher levels of SSS, having stress episodes, and levels of exertion during activities of daily living. Therefore, it is possible that the *COMT* Val158Met variant plays a relevant role in the phenotypic expression of FM. The study of genetic variants would be a helpful tool for better identification and classification of these patients. Given the heterogeneity of FM patients, the identification of standard phenotypes and genetic profiles could help focus personalized medicine and tailored therapies for each patient, in addition to considering gender as a separate category in chronic pain studies.

## Figures and Tables

**Table 1:** Baseline clinical and phenotype features of FM and non-FM participants separated by gender.

	Women				Men			
	Total (*n* = 421)	Non-FM (*n* = 120)	FM (*n* = 301)	*p*	Total (*n* = 25)	Non-FM (*n* = 15)	FM (*n* = 10)	*p*
Age (mean ± SD) *	55.29 ± 10.66	51.57 ± 14.43	56.77 ± 8.28	**<0.001**	52.52 ± 10.85	49.87 ± 12.21	56.50 ± 7.24	0.137
**Place of residence (%)**								
Rural	24.52	33.30	21.00	**0.008**	28.00	20.00	40.00	0.275
Urban	75.48	66.70	79.00		72.00	80.00	60.00	
Drug number (mean ± SD) *	5.42 ± 2.78	1.04 ± 0.20	5.42 ± 2.78	**<0.001**	5.71 ± 1.98	1.00 ± 0.00	5.71 ± 1.98	**<0.001**
Antidepressant (%)	36.50	7.50	54.74	**<0.001**	24.00	0.00	60.00	**0.001**
Benzodiazepines (%)	30.60	6.67	45.79	**<0.001**	12.00	0.00	30.00	**0.030**
Analgesics (%)	48.40	6.67	74.74	**<0.001**	28.00	0.00	70.00	**<0.001**
Others (%)	21.30	43.33	7.37	**<0.001**	12.00	13.33	10.00	0.952
**Polymedication (%)**								
Yes	36.10	0.00	58.90	**<0.001**	28.00	0.00	70.00	**<0.001**
No	63.90	100.00	41.10		72.00	100.00	30.00	
**FSS (%)**								
No problem	23.10	72.50	1.50	**<0.001**	32.00	53.30	0.00	**<0.001**
Mild	17.01	20.80	15.30		32.00	46.70	10.00	
Moderate	56.09	5.80	78.10		32.00	0.00	80.00	
Severe	3.81	0.80	5.10		4.00	0.00	10.00	
**WunSS (%)**								
No problem	18.02	56.70	1.10	**<0.001**	44.00	73.30	0.00	**<0.001**
Mild	18.27	28.30	13.90		12.00	20.00	0.00	
Moderate	49.49	12.50	65.70		32.00	6.70	70.00	
Severe	14.21	2.50	19.30		12.00	0.00	30.00	
**CoSS (%)**								
No problem	25.13	80.80	0.70	**<0.001**	56.00	93.30	0.00	**<0.001**
Mild	18.78	16.70	19.70		8.00	6.70	10.00	
Moderate	54.31	2.50	77.00		36.00	0.00	90.00	
Severe	1.78	0.00	2.60		0.00	0.00	0.00	
Total SSS (0–12) (mean ± SD) *	4.33 ± 2.46	1.18 ± 1.59	5.72 ± 1.14	**<0.001**	3.00 ± 2.81	0.87 ± 0.91	6.20 ± 0.91	**<0.001**
Average VAS (0–10) (mean ± SD) *	2.71 ± 2.51	1.24 ± 1.94	3.30 ± 2.47	**<0.001**	2.08 ± 2.25	1.27 ± 1.53	3.30 ± 2.67	**0.024**
Weak (0–3) (%)	76.26	84.90	72.80	**0.006**	84.00	86.70	80.00	0.452
Medium (4–7) (%)	16.07	13.40	17.10		12.00	13.30	10.00	
Severe (8–10) (%)	7.67	1.70	10.10		4.00	0.00	10.00	
**Rest quality (%)**								
High	22.86	62.20	7.30	**<0.001**	40.00	66.70	0.00	**0.003**
Medium	71.90	31.10	88.00		56.00	33.30	90.00	
Low	5.24	6.70	4.70		4.00	0.00	10.00	
**Stress episode (%)**								
Yes	72.62	21.40	93.20	**<0.001**	56.00	26.70	100.00	**<0.001**
No	27.38	78.60	6.80		44.00	73.30	0.00	
**Borg scale (0–10) (%)**								
Easy or light (0–2)	25.65	44.20	18.30	**<0.001**	16.00	20.00	10.00	**0.006**
Light intensity (3–4)	63.66	26.70	78.40		48.00	20.00	90.00	
Mild intensity (5–6)	7.36	17.50	3.30		20.00	33.30	0.00	
Vigorous intensity (7–10)	3.33	11.70	0.00		16.00	26.70	0.00	
WPI (mean ± SD) *	11.12 ± 3.94	-	11.12 ± 3.94	-	11.20 ± 3.93	-	11.20 ± 3.93	-
WPI + SSS (mean ± SD) *	16.72 ± 4.21	-	16.72 ± 4.21	-	17.40 ± 4.17	-	17.40 ± 4.17	-

* Data are shown as mean ± standard deviation. FSS: fatigue; WunSS: waking unrefreshed; CoSS: cognitive symptoms; SSS: symptom severity scale; VAS: visual analogue scale; WPI: widespread pain index. Statistically significant values are in bold.

**Table 2:** Genotype and allele frequencies in FM and non-FM participants.

	Women			Men		
	Non-FM (*n* = 120)	FM (*n* = 301)	*p*	Non-FM (*n* = 15)	FM (*n* = 10)	*p*
	% (*n*)	% (*n*)		% (*n*)	% (*n*)	
***COMT rs4680***						
G472G (Val158Val)	34.5 (41)	25.9 (78)	0.203	13.3 (2)	30 (3)	0.584
G472A (Val158Met)	46.2 (55)	53.8 (162)		53.4 (8)	40 (4)	
A472A (Met158Met)	19.3 (23)	20.3 (61)		33.3 (5)	30 (3)	
Allele G472 (Val158)	0.57	0.53	0.361	0.40	0.50	**0.045**
Allele A472 (Met158)	0.43	0.47		0.60	0.50	
***OPRM1 rs1799971***						
A118A (Ans40Asn)	70.6 (84)	68.1 (205)	0.879	80 (12)	30 (3)	**0.036**
A118G (Asn40Asp)	27.7 (33)	29.9 (90)		20 (3)	60 (6)	
G118G (Asp40Asp)	1.7 (2)	2 (6)		0 (0)	10 (1)	
Allele A118 (Asn40)	0.84	0.83	0.618	0.90	0.60	**<0.001**
Allele G118 (Asp40)	0.16	0.17		0.10	0.40	

*OPRM1*: µ-opioid receptor 1; *COMT*: catechol-O-methyltransferase. Statistically significant values are in bold.

**Table 3:** Logistic regression model testing the influence of genotypes on the phenotypes of female participants.

	*OPRM1 rs1799971* Dominant Model				*COMT rs4680* Dominant Model			
	Crude		Adjusted by Age		Crude		Adjusted by Age	
	*p*-Value	OR (95% CI)	*p*-Value	OR (95% CI)	*p*-Value	OR (95% CI)	*p*-Value	OR (95% CI)
**FSS**	0.433	-	0.358	-	**0.042**	1.592 (1.02–2.49)	**0.014**	1.010 (1.00–1.02)
**WunSS**	0.787	-	0.837	-	0.061	1.541 (0.98–2.42)	**0.032**	1.008 (1.00–1.02)
**CoSS**	0.475	-	0.425	-	0.126	-	0.068	1.007 (1.00–1.02)
**Total SSS**	0.662	-	0.655	-	**0.010**	1.810 (1.16–2.84)	**0.007**	1.011 (1.00–1.02)
**VAS**	0.224	-	0.201	-	0.639	-	0.590	-
**Rest quality**	0.997	-	0.862	-	**0.047**	1.633 (1.01–2.65)	**0.008**	1.011 (1.00–1.02)
**Stress episode**	0.508	-	0.376	-	**0.013**	1.802 (1.13–2.87)	**0.005**	1.011 (1.00–1.02)
**Borg scale**	0.504	-	0.707	-	**0.013**	2.232 (1.19–4.18)	**0.001**	1.019 (1.01–1.03)
**WPI**	0.105	-	0.083	1.014 (1.00–1.03)	0.869	-	0.993	-
**WPI + SSS**	0.359	-	0.338	-	0.655	-	0.601	-

*OPRM1*: µ-opioid receptor 1; *COMT*: catechol-O-methyltransferase; FSS: fatigue; WunSS: waking unrefreshed; CoSS: cognitive symptoms; SSS: symptom severity scale; VAS: visual analogue scale; WPI: widespread pain index. Statistically significant values are in bold.

**Table 4:** Logistic regression model testing the influence of genotypes on the phenotypes of male participants.

	*OPRM1 rs1799971* Dominant Model				*COMT rs4680* Dominant Model			
	Crude		Adjusted by Age		Crude		Adjusted by Age	
	*p*-Value	OR (95% CI)	*p*-Value	OR (95% CI)	*p*-Value	OR (95% CI)	*p*-Value	OR (95% CI)
**FSS**	**0.050**	6.000 (1.00–35.91)	**0.032**	1.035 (1.00–1.07)	0.835	-	0.866	-
**WunSS**	**0.007**	16.000 (2.17–118.27)	**0.006**	1.053 (1.02–1.09)	0.427	-	0.764	-
**CoSS**	**0.050**	6.000 (1.00–35.91)	**0.033**	1.035 (1.00–1.07)	0.226	-	0.451	-
**Total SSS**	**0.018**	9.333 (1.47–59.48)	**0.015**	1.042 (1.01–1.08)	0.318	-	0.553	-
**VAS**	0.999	-	0.999	-	0.999	-	0.749	-
**Rest quality**	**0.027**	13.500 (1.34–135.99)	**0.029**	1.047 (1.01–1.09)	1.000	-	0.716	-
**Stress episode**	**0.015**	17.857 (1.75–200.00)	**0.016**	1.053 (1.01–1.10)	0.840	-	0.777	-
**Borg scale**	0.185	-	0.148	-	0.417	-	0.596	-
**WPI**	0.501	-	0.378	-	0.501	-	0.492	-
**WPI + SSS**	0.999		0.737		0.999		0.999	

*OPRM1*: µ-opioid receptor 1; *COMT*: catechol-O-methyltransferase; FSS: fatigue; WunSS: waking unrefreshed; CoSS: cognitive symptoms; SSS: symptom severity scale; VAS: visual analogue scale; WPI: widespread pain index. Statistically significant values are in bold.

## Data Availability

The data that support the findings of this study are available on request from the corresponding author.

## Abbreviations

The following abbreviations are used in this manuscript:

FM	Fibromyalgia
OPRM1	μ-opioid receptor 1
COMT	Catechol-O-methyltransferase
PROMs	Patient-reported outcome measures
WPI	Widespread pain index
VAS	Visual analogue scale
SSS	Symptom Severity Scale
FSS	Fatigue levels
WunSS	Waking unrefreshed
CoSS	Cognitive symptoms
RPE	Ratings of perceived exertion
ADL	Activities of daily living
ACR	American College of Rheumatology
LR	Logistic regression
